# Correction: Influences of atmospherics on customer satisfaction and behavioural intentions in the restaurant industry: Evidence from an emerging economy

**DOI:** 10.1371/journal.pone.0324038

**Published:** 2025-05-23

**Authors:** Mananage Shanika Hansini Rathnasiri, Pawan Kumar, Bindu Aggarwal, Kiran Nair, Narayanage Jayantha Dewasiri

There is an error in affiliation 3 for author Bindu Aggarwal. The correct affiliation 3 is: University School of Business Chandigarh University, Mohali-140413, Punjab, India.
