# Supplementary material for: Influences of atmospherics on customer satisfaction and behavioural intentions in the restaurant industry: Evidence from an emerging economy
Source: PLoS One. 2025 Apr 4;20(4):e0319948. doi: 10.1371/journal.pone.0319948 (PMC11970681; doi:10.1371/journal.pone.0319948)
Supplement: Attachment — Submitted filename: PONE-D-24-10577_review.docx [file pone.0319948.s001.docx]

Influences of Atmospherics on Customer Satisfaction and Behavioral Intentions in the Restaurant Industry: Evidence from an Emerging Economy

I think the topic of this study is interesting in that it aims to identify the impacts of atmospherics of fine dining restaurant on customer satisfaction and behavioral intentions. However, the following issues must be addressed:

1. Upon a thorough examination of the entire paper, it becomes apparent that the study lacks a well-developed logical foundation based on theory. Despite collecting the data in New Delhi, the capital of India, there is a noticeable absence of a theoretical contribution. In other words, we cannot find a key theory that explains the theoretical background and contributions of this study compared to previous studies in the introduction and implications section.
2. Why did the authors use the term of configuration-related attributes? The meaning of the term is not clearly understood.
3. Since restaurant attributes consist of four sub-dimensions, the H1 must be presented for each subdimension.
4. Research Instrument and Data should be divided into 1) Instrument, 2) Sampling and data collection in the Methodology section.
5. The authors should make the Results section.
6. What is the difference between Table 4 and Table 5? To explain the measurement model, it may be sufficient to use just one appropriate table.
7. To verify the mediation effect, the authors only need to use one table, so please integrate the tables.
8. The authors need to update more recent papers.
9. Overall, the structure of the manuscript is not systematic, so please revise the structure of it.
10. Other

- The references should be edited by the editorial policy of the journal.
- Overall, the sentence is not concise, so please edit the entire sentence.
- Please check the text carefully for grammar and typos.
- Delete “Source: Authors’ own” below the tables and figures.
- If the authors wish to show Figures of the AMOS analysis results, please correct them.
