# Supplementary material for: Influences of atmospherics on customer satisfaction and behavioural intentions in the restaurant industry: Evidence from an emerging economy
Source: PLoS One. 2025 Apr 4;20(4):e0319948. doi: 10.1371/journal.pone.0319948 (PMC11970681; doi:10.1371/journal.pone.0319948)
Supplement: Attachment — Submitted filename: Response to Reviewers.docx [file pone.0319948.s002.docx]

Influences of Atmospherics on Customer Satisfaction and Behavioral Intentions in the Restaurant Industry: Evidence from an Emerging Economy

Reviewer 01

I think the topic of this study is interesting in that it aims to identify the impacts of the atmospherics of fine dining restaurants on customer satisfaction and behavioural intentions. However, the following issues must be addressed:

1. Upon a thorough examination of the entire paper, it becomes apparent that the study lacks a well-developed logical foundation based on theory. Despite collecting the data in New Delhi, the capital of India, there is a noticeable absence of a theoretical contribution. In other words, we cannot find a key theory that explains the theoretical background and contributions of this study compared to previous studies in the introduction and implications section.

Action Taken: To investigate the relationship between the atmospherics of fine dining restaurants, customer satisfaction and behavioural intentions, we adopted the Stimulus-Organism-Response (SOR) theory proposed by Mehrabian and Russell (1974). The study aims to examine restaurant attributes as stimuli (S), overall customer satisfaction as an organism (O), and behavioural intentions as a response (R). Theoretical contributions have been addressed in the introduction and the implications section. (PP. 2,3, 23)

2. Why did the authors use the term of configuration-related attributes? The meaning of the term is not clearly understood.

Action Taken: The term Configuration-related attributes has been replaced by a more relevant term Spatial Configuration (Lima et al., 2024; Selem et al., 2023) to have a better understanding of its significance in fine dining restaurants. (PP. 5,7, supporting references have been added in the text as well as in the references section).

3. Since restaurant attributes consist of four sub-dimensions, the H1 must be presented for each subdimension.

Action Taken: HI is now being presented for all the four sub-dimensions. (Incorporated on PP. 8).

4. Research Instrument and Data should be divided into 1) Instrument, 2) Sampling and data collection in the Methodology section.

Action Taken: The suggestion has been incorporated and the methodology section has been divided into two Instrument & Data Collection. ((PP. 11,12).

5. The authors should make the Results section.

Action Taken: A separate section for results has been added. (PP. 19,20).

6. What is the difference between Table 4 and Table 5? To explain the measurement model, it may be sufficient to use just one appropriate table.

## Action Taken: Table 5 has been removed now, and the numbering of the tables has now changed from Table 4 onwards. (PP. 16,17, Table 5 is now: Regression Weights Restaurant Atmospherics, Overall Customer Satisfaction and Behavioral Intentions).

7. To verify the mediation effect, the authors only need to use one table, so please integrate the tables.

Action Taken: Tables showing Direct & Indirect mediation effects have been integrated into one table and the Summary table for Mediation Analysis has also been removed to avoid duplicity. (PP. 18).

8. The authors need to update more recent papers.

Action Taken: Added Highlighted in yellow in the references section.

9. Overall, the structure of the manuscript is not systematic, so please revise the structure of it.

Action Taken: The structure of the manuscript has now been revised.

10. Other

- The references should be edited according to the editorial policy of the journal.
- Overall, the sentence is not concise, so please edit the entire sentence.
- Please check the text carefully for grammar and typos.
- Delete “Source: Authors’ own” below the tables and figures.
- If the authors wish to show Figures of the AMOS analysis results, please correct them.

Action Taken: The above suggestions have been incorporated.

**Reviewer 02**

Reviewer Comment 01: The latest papers are not cited.

Action Taken: Thank you for your valuable feedback. We appreciate your suggestion to include the latest papers in our manuscript. In response to your comment, we have thoroughly reviewed recent literature and updated our manuscript accordingly.

Reviewer Comment: 02: English grammar needs to be improved.

Action Taken: Thank you for your insightful comment regarding the English grammar in our manuscript. To address this, we have thoroughly reviewed the text and made necessary corrections to enhance its clarity and readability. We also sought the assistance of a native English speaker to ensure that the language was polished and professional. These improvements have significantly enhanced the quality of our manuscript.

Reviewer Comment: 03: More synthesis with the current literature will make the paper sound in the discussion section.

Action Taken: Thank you for your valuable feedback on the need for more synthesis with current literature in the discussion section. We have carefully revised this section to integrate recent studies and provide a more comprehensive analysis.

Reviewer Comment: 04: Table 10 is not mentioned in the manuscript.

Action Taken: Thank you for pointing out the issue with Table 10. Upon review, we realized that Table 10 is not present in the manuscript and believe that the reference was intended for Table 1. We have now ensured that Table 1 is appropriately mentioned and discussed in the manuscript.

Reviewer Comment: 05: Future research areas not mentioned

Action Taken: Thank you for your insightful feedback regarding omitting future research areas. We agree that outlining future research directions is crucial for advancing the field. In response to your comment, we have added a section on future research areas in the conclusion of our manuscript.
