# Supplementary material for: Influences of atmospherics on customer satisfaction and behavioural intentions in the restaurant industry: Evidence from an emerging economy
Source: PLoS One. 2025 Apr 4;20(4):e0319948. doi: 10.1371/journal.pone.0319948 (PMC11970681; doi:10.1371/journal.pone.0319948)
Supplement: Attachment — Submitted filename: PONE-D-24-10577_review R1.docx [file pone.0319948.s003.docx]

Influences of Atmospherics on Customer Satisfaction and Behavioral Intentions in the Restaurant Industry: Evidence from an Emerging Economy

This manuscript still has a lot to improve, even though the original comments have been revised to some extent. In particular, this study applied the SOR model, but it does not seem to have any theoretical contribution compared to previous studies.

The authors need to simplify several tables and present them as one, but they present too many tables unnecessarily, which leads to a lack of conciseness and clarity in the paper. This is also the case in the main text. Overall, the authors should write concisely.

What does *** mean in Table 4?

There are cases where the table presenting the results of the structural model analysis does not match the contents of the main text.

I do not know what the table explaining the parameters is trying to explain.

The authors should organize and present the results of the AMOS program analysis in Table 6 for the readers instead of presenting them as they are. What does *** mean in Table 6?

7.3. Future Directions -> 7.3. Limitations and Future Research Directions

The references should be edited by the editorial policy of the journal.

Figure 1 should display the four subdimensions of restaurant selection attributes.

I recommend the authors review other papers.

e.g.)

Jun, K., & Yoon, B. (2024). Consumer perspectives on restaurant sustainability: an SOR Model approach to affective and cognitive states. *Journal of Foodservice Business Research*, 1-24.
