# Supplementary material for: Influences of atmospherics on customer satisfaction and behavioural intentions in the restaurant industry: Evidence from an emerging economy
Source: PLoS One. 2025 Apr 4;20(4):e0319948. doi: 10.1371/journal.pone.0319948 (PMC11970681; doi:10.1371/journal.pone.0319948)
Supplement: Attachment — Submitted filename: Response_to_reviewers_auresp_2.docx [file pone.0319948.s004.docx]

**Response to Reviewers**

| 1. | Theoretical contribution of the study | Theoretical contributions have been added on the page no.- 23 |
| --- | --- | --- |
| 2. | There are cases where the table presenting the results of the structural model analysis does not match the contents of the main text. | Corrected on page no. 16,17 & 19 |
| 3. | What does *** mean in Table 4? | p<0.01**inter-construct correlation value) |
| 4. | What does *** mean in Table 6? | p<0.001*** Inter-construct correlation value. |
| 5. | Organize and present the results of the AMOS program analysis in Table 6 for the readers instead of presenting them as they are. | Incorporated on page no. 17 & 18 |
| 6. | Limitations and Future Research Directions | Added on Page no. 23 |
| 7. | Figure 1 should display the four subdimensions of restaurant selection attributes. | Incorporated in the Figure 1 |
| 8. | References should be edited by the editorial policy of the journal | It has been done |
