# Supplementary material for: Influences of atmospherics on customer satisfaction and behavioural intentions in the restaurant industry: Evidence from an emerging economy
Source: PLoS One. 2025 Apr 4;20(4):e0319948. doi: 10.1371/journal.pone.0319948 (PMC11970681; doi:10.1371/journal.pone.0319948)
Supplement: Attachment — Submitted filename: Response_to_Reviewers_auresp_3.docx [file pone.0319948.s005.docx]

**PLOS ONE Decision: Reviewer 3 Comments & Revision- All revisions are highlighted in yellow.**

| **1. It is suggested to change the title as “Investigating the relationships of atmospherics, customer satisfaction andbehavioural intentions: Evidence from the fine dining restaurant”.** | The title has been changed on page no. 1 |
| --- | --- |
| **2. “According to the National Restaurant Association of India, India Food Services Report, 2024, the Indian food industryis estimated to be Rs 5,69,487 crore for FY24. It is expected to grow to 7,76,511 crores by FY28, achieving a CAGR of8.1 per cent, and it will grow with a CAGR of 13.2%”. It is suggested to convert these figures into US dollars.** | Incorporated on page no. 3 |
| **3. P3. Third paragraph, what does “Divan” mean? Please consider global audience, when you discuss or introduce anyterms.** | Incorporated on page no. 3 |
| **4. It is suggested to club the literature review section and operational definition of constructs.** | Incorporated on page no. 7, section 2.6 |
| **5. Table 5, relationships, it is suggested to reorder all the relationships (for example SA. CS, MENU. CS etc.).** | Incorporated on page no. 17 |
| **6. It is suggested to merge the “Findings with “Discussions and Implications”.** | Incorporated on page no. 20 |
| **7. In the discussions and implications section, it is suggested that theoretical implications must be first discussed in detail (which is too short), and then practical implications must be discussed, offering better insights.** | Incorporated on page no. 22 |
| **8. It is suggested to strictly follow the format guidelines of the journal.** | **Guidelines are now being followed** |
| **9. Proof reading is recommended.** | **Proofreading has been done** |

**Reviewer 4 Comment & Revision**

| 1. Kindly elaborate further on the research findings in your discussion. | Incorporated on page no. 20 & 21 |
| --- | --- |
| 2. Please refine your introduction by elaborating on the urgency of the factors and the significance of the topic. | Incorporated on page no. 2 |
